# Post-NAC Microcalcifications in Breast Cancer: Rethinking Surgical Indications in the Era of Precision Oncology

**DOI:** 10.3390/jpm16010049

**Published:** 2026-01-12

**Authors:** Sabatino D’Archi, Beatrice Carnassale, Lorenzo Scardina, Cristina Accetta, Flavia De Lauretis, Alba Di Leone, Antonio Franco, Federica Gagliardi, Stefano Magno, Francesca Moschella, Maria Natale, Alejandro Martin Sanchez, Marta Silenzi, Pierluigi Maria Rinaldi, Gianluca Franceschini

**Affiliations:** 1 Breast Surgery Unit, Department of Woman and Child’s Health and Public Health Sciences, Fondazione Policlinico Universitario A. Gemelli IRCCS, 00168 Rome, Italy; 2 Unit of Radiology and Interventional Radiology, Mater Olbia Hospital, 07026 Olbia, Italy

**Keywords:** breast cancer, neoadjuvant chemotherapy, microcalcifications, residual disease, radiologic–pathologic correlation, breast-conserving surgery, precision surgery, MRI, contrast-enhanced mammography, radiomics

## Abstract

Residual microcalcifications after neoadjuvant chemotherapy (NAC) in breast cancer remain a complex diagnostic and therapeutic challenge. Although NAC has significantly improved pathologic complete response (pCR) rates and transformed surgical approaches, the persistence or evolution of microcalcifications may not accurately reflect residual disease. This discrepancy complicates radiologic interpretation, impacts surgical decision-making, and may lead to overtreatment or unnecessary mastectomies. This review synthesizes current evidence on the radiologic–pathologic correlation of post-NAC microcalcifications, their prognostic value, and their relevance to guiding surgical management in contemporary precision oncology. A narrative review of the literature was performed, focusing on imaging evolution after NAC, pathologic correlations, predictive and prognostic implications, and the role of microcalcifications in defining optimal surgical strategies, ranging from breast-conserving surgery to mastectomy. Emerging contributions from digital breast tomosynthesis, contrast-enhanced mammography (CEM), Magnetic Resonance (MR) and radiomics are also examined. Studies consistently demonstrate that residual microcalcifications are often poor predictors of viable tumor tissue after NAC. Up to half of cases with persistent calcifications may reflect minimal or absent residual invasive cancer, whereas calcifications may also persist in areas of treatment-induced necrosis or fibrosis. Reliance on calcifications alone may therefore lead to unnecessary extensive resections. Conversely, specific morphologic patterns, especially fine pleomorphic or branching calcifications, are more strongly associated with residual malignancy. Advanced imaging and radiomics show promise in improving predictive accuracy. Residual microcalcifications after NAC should not be interpreted as a direct surrogate of residual disease. A multimodal assessment integrating imaging evolution, tumor biology, and treatment response is essential to optimize surgical planning and avoid overtreatment. Precision surgery in the NAC era increasingly requires individualized decision-making supported by advanced imaging and robust radiologic–pathologic correlation.

## 1. Introduction

Neoadjuvant chemotherapy (NAC) has become integral to the management of breast cancer, particularly for aggressive subtypes such as triple-negative and HER2-positive tumors. Beyond improving rates of pathological complete response (pCR), NAC frequently downstages disease, enabling a transition from mastectomy to breast-conserving surgery (BCS) and enhancing both functional and cosmetic outcomes.

In this context, accurate radiologic assessment of treatment response is essential. Among post-treatment findings, the persistence of microcalcifications on mammography (MG) remains one of the most controversial and clinically consequential issues. Traditionally interpreted as indicators of ductal carcinoma in situ (DCIS) or residual invasive disease, persistent calcifications are recommended for complete excision by major guidelines, including the NCCN [1]. However, this paradigm may lead to unnecessarily wide resections or mastectomies in patients otherwise suitable for BCS.

Recent studies challenge the assumption that post-NAC microcalcifications consistently reflect malignancy, reporting that 40–70% of them represent benign post-therapeutic alterations, such as necrosis or fibrosis. Radiologic–pathologic concordance is highly variable, and the positive predictive value of imaging for residual disease remains modest, particularly for DCIS.

As a result, current management practices often risk surgical overtreatment with implications for morbidity and aesthetic outcomes. In light of these uncertainties, a critical reassessment of post-NAC microcalcifications is urgently needed.

This review examines their oncologic relevance, evaluates the performance of advanced imaging modalities, and discusses future strategies, grounded in radiomics, artificial intelligence, and molecular profiling, for guiding safe, individualized surgical de-escalation.

## 2. The Diagnostic Role of Breast Microcalcifications

Microcalcifications represent one of the primary radiological manifestations of breast cancer, particularly DCIS, but they may also be present in invasive lesions or benign conditions. They are defined as calcium deposits smaller than 1 mm, typically detected by conventional MG, often in the absence of palpable or symptomatic abnormalities [2].

### 2.1. Radiological Characterization

The classification of microcalcifications according to the BI-RADS (Breast Imaging Reporting and Data System) system is based on morphology (amorphous, pleomorphic, branching, fine linear, etc.) and distribution (clustered, segmental, linear, diffuse). Pleomorphic and segmental microcalcifications are considered highly suspicious for malignancy and are frequently associated with DCIS or invasive carcinoma with an intraductal component [3].

MG is the first-line imaging modality for their detection due to its high sensitivity in visualizing even tiny calcifications. However, its specificity is limited, as similar findings can also be observed in sclerosing adenosis, fat necrosis, fibrosis, or post-treatment changes [4].

### 2.2. Histological Correlation

From a histopathological standpoint, microcalcifications may reflect a broad spectrum of conditions, ranging from benign lesions (e.g., calcified fibroadenomas, fat necrosis, stromal calcifications) to preinvasive or invasive diseases. In the context of NAC, the histological significance of microcalcifications is particularly complex: studies have shown that many persistent post-treatment calcifications are not associated with residual tumor cells but rather with intraductal necrosis, fibrosis, or scarring [5,6].

According to Kim et al., in a study of over 320 patients who received NAC, 44.8% of residual microcalcifications did not correlate with residual tumor upon pathological examination [7]. Similarly, An et al. reported poor concordance between the extent of microcalcifications on MG and the histological size of the residual tumor, suggesting a potential overestimation of disease burden by imaging [8].

Moreover, Um et al. observed that MG tends to overestimate disease extent relative to magnetic resonance imaging (MRI), particularly in hormone receptor-negative subtypes, in which calcifications often represent necrotic remnants rather than active disease [9].

These data highlight that while the presence of microcalcifications is an essential diagnostic indicator, it should not, in itself, be considered evidence of an active tumor after NAC, but rather be interpreted within a broader clinical, radiological, and biological context.

## 3. The Impact of Neoadjuvant Chemotherapy on Breast-Conserving Surgery

NAC was initially introduced for the treatment of locally advanced and inoperable breast cancer. However, with the improvement of pharmacological regimens and the molecular classification of breast cancer, its use has extended to operable tumors as well, to reduce tumor size and facilitate a conservative surgical approach without compromising oncologic safety [10,11].

Several randomized studies and meta-analyses have shown that NAC enables, in a significant proportion of cases, the conversion from mastectomy to conservative surgery without increasing the risk of local recurrence, provided that adequate surgical margins are achieved [12,13]. The pCR rate is a significant prognostic marker, associated with better local control and increased disease-free survival, especially in HER2-positive and triple-negative tumors [14].

Appropriate selection of patients who may benefit from conservative surgery requires a careful radiological assessment after NAC. MG, ultrasound, and especially MRI are used to monitor treatment response and define the extent of residual disease. In this setting, MRI has demonstrated superior sensitivity in predicting pCR and in evaluating the residual tumor pattern [15,16].

In the case of nodular lesions, it is relatively easy for radiologists to perform accurate restaging and for surgeons to perform safe resection. This is possible even in cases of complete radiological response, thanks to the placement of a marker clip before treatment, or in cases of partial response, due to residual tumor visibility [17]. However, when residual microcalcifications are present after NAC, they are often considered a relative contraindication to breast-conserving surgery, prompting wider resections or mastectomy, even in patients with a complete pathological response.

The lack of correlation between microcalcifications and residual tumor raises significant doubts regarding current surgical strategies, especially in the context of precision oncology. This discrepancy underscores the need for a critical reassessment of surgical indications when microcalcifications persist after NAC.

International guidelines—including those from the NCCN—still recommend the complete removal of post-NAC microcalcifications, based on the assumption that they may represent areas of DCIS or residual invasive disease [1]. While this conservative approach is precautionary, it is increasingly challenged by recent literature, which highlights a growing disconnect between radiologic findings and histopathological reality.

## 4. Post-NAC Microcalcifications and Pathologic Response: How Predictive Are They?

Multiple studies have shown that residual microcalcifications after NAC do not reliably correlate with residual tumor and that the role of microcalcifications varies across molecular subtypes.

Golan et al. [18] provided essential evidence correlating changes in microcalcification patterns with treatment response. In their retrospective series, 42.6% of patients demonstrated a decrease in microcalcifications following NAC, correlating with higher pCR rates (59% vs. 20% when microcalcifications remained stable, *p* = 0.009). However, they also observed numerous complete responders whose microcalcifications were unchanged, demonstrating that mammographic persistence does not equate to residual disease. The study also analyses the role of microcalcifications across molecular subtypes, showing that HER2-positive and triple-negative subtypes are more likely to exhibit fewer microcalcifications and achieve pCR than luminal subtypes. This suggests that persistent calcifications in aggressive subtypes should be interpreted cautiously and not automatically considered residual malignancy.

Complementary evidence from Bae et al. demonstrated that in HR-negative and HER2-positive subtypes, residual microcalcifications after NAC were often benign, with up to 96% of HR-negative complete responders showing no invasive tumor despite persistent microcalcifications. Conversely, in HR-positive tumors, residual calcifications were more frequently associated with DCIS or low-volume invasive disease, supporting a subtype-adapted surgical approach [19].

Lee et al. [20] examined 144 post-NAC breast specimens with diffuse microcalcifications and reported that although 80% retained microcalcifications, most did not contain invasive carcinoma. The authors nevertheless recommended complete excision for oncological safety, given the coexistence of in situ carcinoma in a minority of cases.

Riordan Azam et al. [5] analyzed 186 patients treated with NAC. They found that in 25.2% of cases, post-treatment microcalcifications did not contain any tumoral component (neither in situ nor invasive) at final pathological examination. Additionally, the size of calcifications assessed on MG overestimated actual tumor extent in over 57% of cases—except in triple-negative tumors, where the correlation was stronger (R^2^ = 83%).

Similar results were reported by An et al., who showed that MG had low accuracy in predicting residual disease when microcalcifications were present. In over 44% of cases, the microcalcifications were not associated with residual tumor cells. Their study compared MG and MRI and found that although MG had a sensitivity of 82%, its specificity was very low (25%), leading to a high rate of false positives [8].

Felicano et al. [6] supported these findings: among patients with residual microcalcifications on MG, pCR was achieved despite their presence, with a strong correlation between MRI enhancement disappearance and pCR (*p* < 0.0001), whereas persistent microcalcifications were not significantly associated with active disease.

One of the most comprehensive contemporary analyses, conducted by Allotey et al., reviewed over five years of institutional data from the National Health Service (NHS) Grampian Health Board. Among women who underwent NAC, the presence of residual microcalcifications was not statistically significantly associated with pCR (*p* = 0.763) and did not affect recurrence or survival (HR for recurrence = 2.599, *p* = 0.393; HR for overall survival = 1.362, *p* = 0.801). The authors concluded that “the predictive and prognostic significance of residual microcalcifications remains to be proven,” recommending that surgical excision be based on individualized oncologic risk rather than radiological persistence alone [21].

The clinical consequences of this overestimation are significant. In practical terms, the presence of microcalcifications is often interpreted as an indication for mastectomy or wide excisions, negatively impacting morbidity, cosmetic outcomes, and quality of life. Jochelson et al. [16] reported that 20% of patients who underwent mastectomy after NAC did so solely due to the presence of residual microcalcifications, findings that were not confirmed as active disease on histologic examination.

Um et al. [9] in a multicentric study of 151 patients, demonstrated that MG systematically overestimates the size of residual tumors, with a high risk of excessive surgery when decisions are based solely on the presence of microcalcifications. Their study found that MRI correlated better than MG in predicting tumor size (intraclass correlation coefficient, ICC 0.769 vs. 0.651). For the HR+/HER2− subtype, MG showed a higher correlation than MRI (ICC = 0.747 vs. 0.575). In the HR− subtype, MRI showed a stronger correlation with pathology (ICC = 0.939 vs. 0.750), whereas MG tended to overestimate tumor size (ICC = 0.543 vs. 0.479). Post-NAC residual microcalcifications on MG have a lower correlation with residual tumor size than MRI. In the HR+/HER2− subtype, the extent of preoperative calcifications may not be a reliable indicator of residual tumor burden after NAC.

In a multicentric study including 323 patients, Kim et al. reached similar conclusions. Stratifying patients by pCR status and microcalcification presence, they found that long-term outcomes were determined by pCR status rather than calcification persistence. Even in cases of residual microcalcifications after pCR, there was no statistically significant increase in regional or distant recurrence (*p* = 0.486). Their results support the notion that microcalcifications may persist as post-treatment sequelae rather than tumor indicators [7].

From comparisons among these studies in the recent literature, it emerges that the isolated presence of post-NAC microcalcifications should not, in itself, be considered a contraindication to breast-conserving surgery in the absence of other signs of residual disease. Therefore, they demonstrate a difference in the correlation between microcalcifications and residual tumor across the various tumor subtypes (Table 1).

### Subtype-Specific Implications of Post-NAC Microcalcifications for Surgical Planning

The clinical significance of persistent microcalcifications after neoadjuvant chemotherapy (NAC) is increasingly recognized as being strongly modulated by tumor biology, rather than representing a uniform radiologic surrogate of residual disease. Accumulating evidence suggests that surgical decision-making based solely on mammographic persistence risks oversimplification and potential overtreatment, and that molecular subtype should serve as a central organizing principle in the interpretation of post-NAC microcalcifications and subsequent surgical planning.

In triple-negative and HER2-positive breast cancers, NAC is associated with high rates of pathological complete response (pCR) and profound treatment-induced changes within the tumor bed. Across multiple retrospective and prospective series, persistent microcalcifications in these subtypes frequently correspond to benign post-therapeutic sequelae—such as necrosis, fibrosis, or dystrophic calcifications—rather than viable residual tumor [5]. In this biological context, contrast enhancement on MRI closely reflects residual invasive disease, as angiogenesis and cellular viability remain the dominant determinants of post-treatment enhancement patterns. Accordingly, several studies have demonstrated that the absence of residual enhancement on MRI strongly correlates with pCR in HR-negative and HER2-positive tumors, even when microcalcifications persist on mammography [8,19]. These findings support a conservative surgical strategy in this subgroup, indicating that breast-conserving surgery should not be excluded solely on the basis of persistent calcifications when complete functional imaging response and radiologic–pathologic concordance are present.

By contrast, hormone receptor-positive breast cancers exhibit a distinct post-NAC biological behavior. In these tumors, residual disease more commonly consists of DCIS or low-volume invasive carcinoma characterized by lower proliferative activity and reduced neoangiogenesis. As a result, residual DCIS frequently lacks contrast enhancement (non-enhancing DCIS), representing a well-documented limitation of MRI in the post-NAC setting [22,23]. In this subtype, persistent microcalcifications are therefore more likely to retain oncologic significance, particularly when associated with suspicious morphologic patterns such as fine linear, branching, or pleomorphic calcifications with segmental or ductal distribution. In such cases, reliance on MRI alone may underestimate residual disease extent and potentially compromise oncologic safety.

Within HR-positive tumors, contrast-enhanced mammography (CEM) assumes a particularly important role. By enabling simultaneous assessment of calcific morphology and vascular activity within a single acquisition, CEM provides incremental diagnostic information that is especially valuable when microcalcifications persist without MRI enhancement. Several studies have shown that the absence of enhancement on CEM in areas of residual microcalcifications is associated with a high likelihood of benign post-treatment changes, whereas persistent enhancement correlates strongly with residual DCIS or invasive carcinoma [24,25,26]. This combined morphologic–functional capability makes CEM a critical tool for refining surgical extent in HR-positive disease, allowing surgeons to distinguish patients who may safely undergo conservative or limited excision from those in whom targeted removal of the calcification field remains warranted.

**Figure 1:**
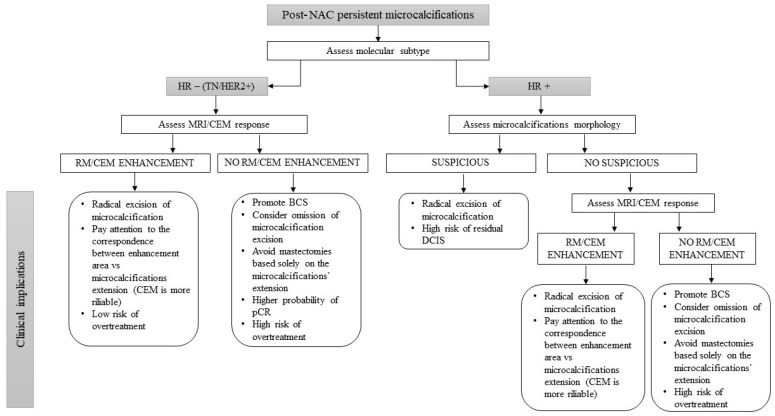
Decision flow-chart for surgical planning in patients with residual microcalcifications after NAC.

Taken together, these subtype-specific patterns clarify why MRI is optimally suited to guide surgical de-escalation in triple-negative and HER2-positive tumors, where enhancement reliably reflects residual disease biology, while CEM plays a pivotal role in hormone receptor-positive cancers, where non-enhancing DCIS represents the principal diagnostic and surgical challenge. Integrating molecular subtype, calcification morphology, and advanced imaging response into a unified, multidisciplinary decision-making framework is therefore essential to avoid both overtreatment in aggressive subtypes with complete response and undertreatment in biologically indolent tumors with persistent in situ disease. This biology-driven approach provides the foundation for individualized surgical planning in the era of precision oncology (Figure 1).

## 5. Evaluating the Accuracy of MRI and CEM in Differentiating Benign Microcalcifications from Residual Malignancy

### 5.1. MRI and Post-NAC Microcalcifications

Several studies confirm that MRI is the most sensitive tool for evaluating response to NAC, particularly for detecting residual invasive disease, due to its ability to assess enhancement patterns linked to tumor neovascularization; however, its sensitivity often decreases in the presence of residual microcalcifications. An et al. analyzed 29 patients with residual microcalcifications after NAC and found that 44.8% of these were not associated with residual malignancy at pathology. Agreement with histopathologic residual tumor size was moderate for MRI (concordance correlation coefficient, CCC = 0.566) and poor for MG (CCC = 0.196). Subtype analysis revealed that MRI was most accurate in triple-negative and HER2-positive tumors (CCC up to 0.8584), whereas performance was lower in ER-positive subtypes [8].

These findings are consistent with Zhu et al., who reported minimal correlation between residual microcalcifications and pathological tumor extent (ICC: 0.097), while MRI showed significantly higher correlation (ICC: 0.771) [27].

Um et al. [9] included 151 patients in their study and found that, for the HR+/HER2− subtype, MG showed a higher correlation than MRI (ICC = 0.747 vs. 0.575). In the HR− subtype, MRI showed a stronger correlation with pathology (HR−/HER2+ or triple-negative (TN), ICC = 0.939 vs. 0.750), whereas MG tended to overestimate tumor size (HR−/HER2+ or TN, ICC = 0.543 vs. 0.479). Overall, MRI performed better than MG in estimating residual tumor size, especially in triple-negative and HER2-positive subtypes (ICC: 0.939 vs. 0.543).

Kim et al. [28] examined 96 post-NAC patients and found that 38.5% of residual microcalcifications were pathologically benign, whereas MRI correlated more closely with residual tumor size (ICC = 0.709) than mammography (ICC = 0.365). The study also emphasizes the influence of molecular subtype on imaging accuracy; correlation was highest in HR+/HER2+ and HR−/HER2+ subtypes (ICC up to 0.925).

A meta-analysis by Marinovich et al. [22], including 44 studies (2050 patients), confirmed an overall area under the curve (AUC) of 0.88 for MRI in detecting residual tumor after NAC—significantly higher than MG. However, MRI showed reduced sensitivity for non-enhancing DCIS, with pooled specificity dropping to 0.61 when DCIS was included in the pCR definition.

Jochelson et al. [16] conducted a pivotal study evaluating the clinical relevance of residual microcalcifications after NAC in patients with locally advanced breast cancer, focusing on cases in which MRI showed no residual contrast enhancement. Their results showed that, in the majority of patients, the absence of MRI enhancement was associated with pCR. However, this correlation was not absolute: a subset of patients exhibited residual DCIS or minimal foci of viable tumor cells within areas of persistent calcifications despite the lack of enhancement.

More recently, Bae et al. [19] conducted one of the most comprehensive retrospective analyses to date exploring the relationship between residual microcalcifications, MRI response, and pathological outcomes following NAC. Presented at the 2023 San Antonio Breast Cancer Symposium, the study included 1078 patients with breast cancer who underwent NAC; among them, 518 patients (48%) exhibited residual microcalcifications on post-NAC MG.

The authors stratified patients according to hormone receptor status and MRI response to determine the clinical significance of these microcalcifications. Among those who achieved a complete MRI response, the pCR rate was 96.1% in HR− tumors and 64.3% in HR+ tumors. Conversely, among patients without complete MRI response, pCR rates fell to 50.8% in HR− and 19.3% in HR+ subtypes.

These results indicate that in HR-negative patients who achieve complete MRI response, residual microcalcifications are frequently non-malignant and may not represent viable tumor tissue. Consequently, aggressive surgical excision in this subset could be safely avoided. In contrast, in HR+ disease, residual microcalcifications are more likely to harbor malignancy, warranting surgical removal to ensure oncological safety.

However, despite its recognized sensitivity for invasive carcinoma, MRI remains limited in detecting non-enhancing DCIS. A systematic review and meta-analysis by Ploumen et al. found that up to 43% of residual DCIS lesions failed to enhance on MRI, potentially leading to underestimation of residual disease and incomplete surgical excision [23].

Taken together, these findings highlight a crucial nuance: while MRI remains the reference standard for evaluating residual invasive disease, its sensitivity decreases markedly in non-enhancing lesions, particularly microcalcifications. Therefore, integrating CEM—which simultaneously evaluates calcific morphology and vascular enhancement—may substantially improve diagnostic accuracy and guide more balanced, personalized surgical decision-making.

### 5.2. CEM and Post-NAC Microcalcifications

CEM is an innovative technique that integrates the visualization of microcalcifications and contrast enhancement into a single acquisition. This makes it particularly well-suited for post-NAC assessment, especially in cases with isolated microcalcifications. It is often preferred in the preoperative setting since, unlike MRI, which is performed in a different breast position, CEM is more reliable in detecting contrast uptake in the area of microcalcifications.

Barra et al. [29] reported that CEM has a sensitivity of 93% for detecting residual disease, while maintaining the ability to visualize microcalcifications, showing better correlation with pathology than MRI (CCC: 0.7 vs. 0.4) and higher specificity (87.5% vs. 75%).

Iotti et al. [25] conducted a prospective study involving 46 patients with breast cancer who underwent NAC and were subsequently evaluated with CEM. Among patients with residual microcalcifications on MG after NAC, 40.9% (9/22) had no enhancement on CEM. Of the non-enhancing microcalcification cases, 77.8% (7/9) were confirmed as non-tumoral on final pathology (necrosis, fibrosis, or stromal calcifications), suggesting a high negative predictive value of absent enhancement for excluding residual disease. In contrast, all enhancing areas (13/13) on CEM correlated with residual invasive or in situ carcinoma, resulting in a positive predictive value (PPV) of 100% for CEM enhancement in this cohort. CEM showed better concordance with pathology in estimating tumor size (CCC = 0.91) compared to conventional mammography (CCC = 0.68) and was non-inferior to MRI (CCC = 0.89). CEM successfully visualized all residual microcalcifications, providing a combined morphological and functional assessment that is not always possible with MRI, particularly in non-enhancing DCIS.

Savaridas et al. [26] specifically explored whether the addition of tomosynthesis and CEM could enhance the prediction of NAC response. Their analysis revealed that when enhancement and calcifications were considered together, diagnostic accuracy significantly improved. The intraclass correlation coefficient (ICC) for CEM in assessing residual DCIS was 0.69, higher than MRI (ICC = 0.66) for invasive components. This suggests that CEM may offer superior characterization of microcalcification-related disease, especially when enhancement is present.

Bernardi et al. [24] confirmed the utility of CEM as a comparable alternative to MRI, particularly when MRI is contraindicated or unavailable. While their primary focus was on tumor sizing, the authors emphasized the advantage of CEM in concurrently evaluating enhancement and calcifications, which is particularly useful for post-NAC surgical planning in cases with indeterminate microcalcifications. In their prospective study, they found that CEM and MRI provided comparable estimates of residual tumor size (mean difference <1 mm vs. pathology) and that CEM represented a viable alternative to MRI when MRI was contraindicated or not tolerated.

Finally, Patel et al. [30] further supported these findings, noting that CEM achieved 95% sensitivity and high concordance with pathology. While the study did not isolate microcalcifications as a separate subgroup, it reinforced the notion that enhancement is a stronger predictor of residual disease than calcifications alone, validating the role of CEM in guiding surgical decision-making when calcifications persist post-treatment.

### 5.3. Integrated Considerations

Overall, available data suggest that although MRI remains the reference standard for evaluating invasive disease, it is limited in assessing non-enhancing microcalcifications. CEM, with its ability to simultaneously evaluate calcific morphology and vascular activity, offers a significant advantage in surgical risk stratification—especially in post-NAC cases with isolated microcalcifications. Sequential or selective integration of both modalities within a multidisciplinary setting can optimize therapeutic strategies, reduce unnecessary interventions, and improve candidate selection for conservative surgery. In particular, the systematic integration of CEM into the post-NAC preoperative work-up is pivotal for patients with isolated microcalcifications and no MRI enhancement. Looking forward, the use of combined imaging approaches and multidisciplinary decision-making algorithms may help reduce overtreatment, ensure oncological safety, and enhance eligibility for breast-conserving surgery. From a practical standpoint, MRI should be considered the reference modality for assessing residual invasive disease after NAC, particularly in triple-negative and HER2-positive tumors. However, in cases of isolated persistent microcalcifications without MRI enhancement—especially in HR-positive disease—CEM should be prioritized, as it allows concurrent evaluation of calcific morphology and vascular activity. A comparative summary of studies about the accuracy of MRI and CEM in evaluating post-NAC microcalcifications is shown in Table 2.

Future challenges will include the prospective validation of patient selection protocols, the development of radiological and molecular biomarkers, and the integration of artificial intelligence into oncological risk stratification.

### 5.4. Management of Discordant Findings

Discordance between imaging modalities—such as persistent microcalcifications on mammography despite complete response on MRI—or between imaging findings and pathological assessment represents a common and clinically challenging scenario in the post-NAC setting. Rather than being resolved by any single modality, such discordance should prompt a structured reassessment that includes verification of spatial concordance between the calcification field, the clipped tumor bed, and any areas of contrast enhancement. Tumor biology plays a central role in this interpretation: in triple-negative and HER2-positive tumors with complete functional imaging response, persistent calcifications are more likely to reflect benign treatment-related changes, whereas in hormone receptor-positive disease, the possibility of residual non-enhancing DCIS remains clinically relevant. In cases of isolated microcalcifications without MRI enhancement—particularly in HR-positive tumors—contrast-enhanced mammography may provide additional risk stratification by correlating calcific morphology with vascular activity. When discordance persists after targeted reassessment, surgical planning should be finalized within a multidisciplinary discussion, balancing oncologic safety with the avoidance of unnecessary extensive resections and clearly documenting the rationale for any de-escalated approach.

## 6. Exploring the Future: The Role of Radiomics, Artificial Intelligence, and Biomarkers

In the evolution of precision oncology, three technological domains are reshaping the landscape of surgical decision-making in breast cancer patients treated with NAC: radiomics, artificial intelligence (AI), and molecular biomarkers. These approaches aim to convert imaging and biological data into quantitative, predictive models capable of distinguishing benign post-therapeutic changes (e.g., residual microcalcifications without viable tumor) from clinically significant residual disease. By integrating advanced computational methods and tumor biology, these tools offer a pathway toward personalized and conservative surgery.

### 6.1. Radiomics in the Assessment of Residual Microcalcifications

Radiomics enables the extraction of hundreds of quantitative imaging features—such as shape, texture, intensity, and temporal variation—that can characterize tissue composition beyond human perception. In post-NAC imaging, radiomics may capture microstructural differences between benign and malignant calcifications, potentially distinguishing necrotic fibrosis from residual DCIS.

Although direct studies of microcalcifications post-NAC remain limited, emerging radiomic frameworks show promise. Fan et al. demonstrated that dynamic contrast-enhanced (DCE)-MRI radiomic features predicted NAC response with an AUC of up to 0.92, which improved to 0.96 when molecular subtype data were included [31].

Similarly, Kontopodis et al. proposed a temporal radiomics approach, demonstrating that MRI-based features could predict response after a single chemotherapy cycle [32].

Applying this to microcalcifications, radiomic descriptors such as entropy, fractal dimension, and gray-level nonuniformity extracted from mammography or CEM could quantify the heterogeneity and organization of calcifications. This might help distinguish benign dystrophic or treatment-induced calcifications from residual DCIS, which typically exhibit higher textural disorder and greater vascular correlation.

In the future, radiomic “fingerprints” combining calcific morphology, spatial clustering, and local enhancement metrics from MRI or CEM could serve as non-invasive surrogates of histopathologic risk—potentially replacing biopsy in selected patients.

Beyond their established role in predicting global response to neoadjuvant chemotherapy, radiomics and artificial intelligence may offer a specific and complementary contribution to the interpretation of persistent post-NAC microcalcifications, addressing a diagnostic gap that conventional visual assessment alone cannot fully resolve. In particular, these technologies may enable a shift from a purely morphology-based interpretation of calcifications toward a quantitative, biology-informed characterization of the treated tumor bed.

In the post-NAC setting, persistent microcalcifications may arise from fundamentally different underlying processes—namely, benign treatment-induced changes versus residual in situ disease—yet these entities often appear similar on standard mammographic evaluation. Radiomic analysis of mammography or contrast-enhanced mammography can interrogate subtle differences in the spatial organization, internal complexity, and clustering behavior of calcifications, which are likely to reflect distinct microstructural substrates. This quantitative profiling may therefore help identify calcification patterns consistent with post-therapeutic fibrosis or necrosis, rather than those associated with residual ductal epithelial proliferation.

Importantly, when radiomic features derived from calcification morphology are combined with enhancement-related parameters on CEM or MRI, AI-based models can integrate morphologic, functional, and clinical information within a unified predictive framework. In this way, radiomics and AI do not replace established imaging criteria but rather refine risk stratification when conventional imaging yields equivocal findings, such as isolated persistent microcalcifications without clear contrast enhancement.

In the context of surgical decision-making, this approach is particularly relevant for supporting selective de-escalation strategies by identifying patients in whom residual microcalcifications are unlikely to harbor clinically meaningful disease and who may therefore safely avoid extensive resection. Although these applications remain investigational and require prospective validation, their integration into multidisciplinary workflows represents a logical extension of precision oncology principles to one of the most debated imaging findings in the neoadjuvant setting.

### 6.2. Artificial Intelligence and Machine Learning

Artificial intelligence (AI) and deep learning (DL) represent the next step toward fully automated image interpretation. AI can integrate radiomic, clinical, and biological variables, learning complex relationships between imaging signatures and pathology outcomes—particularly relevant for residual microcalcifications where morphology alone is insufficient.

AI-driven MRI and CEM models have already shown excellent performance in predicting pCR and residual cancer burden (RCB). For example, Huang et al. developed a 3D U-Net-based system trained on more than 2200 NAC patients, achieving an AUC > 0.91 for predicting tumor regression patterns and residual disease across all subtypes [33].

Likewise, Li et al. created a multitask AI model for early prediction of RCB, distinguishing complete responders from partial or resistant cases with an AUC of up to 0.97 [34].

Translating these models to microcalcifications, AI can integrate mammographic features, CEM enhancement data, and MRI non-enhancing patterns to classify whether residual calcifications are benign post-treatment sequelae or harbor DCIS.

Furthermore, Reid et al. demonstrated that AI-powered 3D visualization platforms based on MRI data improved surgeon–radiologist concordance and patient communication in preoperative planning [35]. Similar interactive systems could map the distribution and vascularity of residual microcalcifications, aiding in targeted resection when necessary.

### 6.3. Molecular Biomarkers and Radiogenomics

A growing body of research supports integrating molecular biomarkers with imaging features—termed radiogenomics—to better interpret post-NAC findings.

Yin et al. demonstrated that merging MRI-based radiomics with genomic data from circulating exocrine bodies accurately predicted NAC response, marking an early success of AI-driven radiogenomics [36].

More recently, Zhang et al. developed a radiopathomic model combining imaging, histopathologic, and clinical features, reaching an AUC of 0.93 for predicting pCR [37].

On the biological front, biomarkers such as TILs, Ki-67, and genomic recurrence scores (e.g., Oncotype DX, MammaPrint) are increasingly linked to imaging response. Iwamoto et al. emphasized that integrating these signatures with AI-based analysis could standardize the evaluation of immune response and residual tumor aggressiveness [38].

Radiogenomic models could thus determine whether residual calcifications correspond to low-proliferation, genomically quiescent tissue or high-risk residual disease, refining post-NAC surgical decision-making.

### 6.4. Clinical Implications: Toward Safe De-Escalation

The integration of radiomics, AI, and biomarkers may redefine management of post-NAC microcalcifications by identifying patients with benign post-therapeutic calcifications and complete radiologic response who may avoid resection and undergo surveillance instead, detecting discordant cases—calcifications harboring residual DCIS despite absent enhancement on MRI—through combined morphologic and vascular assessment, supporting multidisciplinary decision-making, providing objective, data-driven risk stratification and enhancing eligibility for breast-conserving surgery without compromising oncologic safety.

This triad of technologies may help resolve one of the key clinical uncertainties in breast oncology—whether or not to excise residual microcalcifications—while reducing overtreatment and optimizing quality of life.

Despite these advances, several limitations must be addressed before clinical adoption: standardization of radiomic parameters and feature reproducibility across imaging systems and centers; external, multicenter validation of AI algorithms, particularly for microcalcification analysis; interpretability and explainability of AI models to ensure clinical trust; development of prospective trials evaluating the safety and outcomes of AI- or radiomics-guided surgical de-escalation.

## 7. Conclusions

The era of neoadjuvant therapy has triggered a paradigmatic shift in breast cancer surgery. The aim is no longer merely the radical removal of the tumor, but rather the achievement of a balance between oncological effectiveness and the preservation of the natural breast shape. The possibility of surgical de-escalation now represents one of the most advanced frontiers in breast surgery. Indeed, this evolution has prompted questioning the necessity of surgery in cases of complete radiological response, with image-guided vacuum-assisted excision (VAE) considered a potential alternative to surgical excision in patients who achieve pCR. However, the persistence of residual microcalcifications after NAC remains a frequent obstacle to de-escalation surgery.

Current international guidelines continue to recommend complete excision of post-neoadjuvant microcalcifications as a standard precautionary approach. Nevertheless, accumulating evidence underscores the importance of an individualized, biology- and imaging-informed interpretation of these findings in carefully selected patients. Accordingly, while emerging data support the feasibility of more tailored surgical strategies, it must be acknowledged that the existing literature is primarily limited by small sample sizes, heterogeneous study designs, and a predominance of retrospective analyses, with a relative paucity of prospective, externally validated data. As a result, high-quality prospective studies and standardized validation are still required before any de-escalation approach can be safely translated into routine clinical practice.

Cumulative evidence indicates that residual microcalcifications after NAC do not possess prognostic or predictive value, and their presence alone should not determine the indication for mastectomy or wide excision. Studies such as those by Allotey [21], Kim [7], and Azam [5] consistently demonstrate poor correlation between microcalcifications and viable tumor tissue, as well as the MG tendency to overestimate disease extent. Nevertheless, some studies, such as those by Lee et al. [18], advise caution in patients with luminal tumors or diffuse microcalcifications, due to the possible presence of microscopic foci of DCIS. Therefore, the decision whether to excise or conserve should be based on integrating predictive pathological factors (such as biological subtype, histologic grade, and treatment response), rather than on radiological appearance alone. Overall, the literature converges on the need for personalized surgical planning. The persistence of post-NAC microcalcifications should not automatically exclude breast-conserving surgery when pathological and clinical data suggest a complete response. In fact, surgical excision driven solely by the persistence of microcalcifications carries a tangible risk of overtreatment, with up to 20–25% of mastectomies reported in the literature being performed for calcifications later proven benign [5,16]. Importantly, these figures derive from study-level retrospective analyses and should not be interpreted as a pooled estimate; definitions of “unnecessary” mastectomy varied across studies but consistently referred to procedures driven by persistent microcalcifications in the absence of residual invasive disease on final pathology. Embracing precision surgery by integrating tumor biology, imaging response, and multidisciplinary evaluation has the potential to reduce unnecessary radical procedures, minimize morbidity, and preserve quality of life without compromising oncologic safety.

A rational de-escalation based on these parameters can ensure oncological safety while minimizing surgical morbidity. In this context, advanced functional imaging techniques such as MRI and CEM can provide crucial support for accurate preoperative assessment.

Advanced imaging modalities, including MRI, contrast-enhanced mammography, tomosynthesis, and radiomics-driven analyses, offer substantial potential to refine the prediction of residual disease and to enhance radiologic–pathologic correlation.

Future efforts should focus on developing standardized imaging criteria, validating radiomic signatures and integrating multimodal data into clinical decision-support algorithms. Such strategies could substantially reduce variability in surgical practice and promote a more individualized, conservative approach when oncologically appropriate.

Ultimately, reinterpreting the role of post-NAC microcalcifications through a precision oncology lens has the potential to optimize surgical management, minimize unnecessary resections, and improve both oncologic safety and quality of life for patients undergoing treatment for breast cancer.

## Figures and Tables

**Table 1:** Comparative summary of studies on post-NAC microcalcification and surgical implications.

Study (Year)	Patients (n)	Benign Microcalcification (%) *	Molecular Subtype Differences	Key Findings
Jochelson et al. (2015) [16]	60	Not reported	Not analyzed; study focused on imaging–pathology discordance	80% (12/15) unnecessary mastectomies due to imaging later proven benign
Golan et al. (2016) [18]	47	Not reported	HR− showed more frequent decreases in microcalcifications and higher pCR rates than HR+ subtypes	Decrease in microcalcifications correlated with higher pCR; persistence did not indicate residual disease
An et al. (2017) [8].	29	44.8%	MRI accuracy is higher in HR−; MG overestimated extent in HR+.	MG overestimated tumor size; MRI was a more accurate predictor.
Um et al. (2018) [9]	151	Not reported	HR− subtypes better assessed by MRI; HR+ tumors better estimated by MG	MRI correlated better with tumor size than MG (ICC ^†^ 0.769 vs. 0.651).
Lee et al. (2022) [20]	144	Not reported	No molecular subtype analysis performed.	Most microcalcifications are benign, but DCIS may coexist; authors recommend excision for oncologic safety.
Felicano et al. (2017) [6]	90	Not reported	Not specified, but MRI response correlated with HR and HER2 status.	pCR was achieved in 9/10 patients, with a strong correlation between the disappearance of MRI c.e. and pCR (*p* < 0.0001).
Azam et al. (2023) [5]	186	25.2%	HR− subtypes showed higher pCR despite persistent calcifications.	MG overestimates disease burden; supports a conservative approach.
Allotey et al. (2025) [21]	42	Not reported	No molecular subtype analysis performed.	No correlation between calcifications and pCR or survival; supports individualized surgical decision-making.
Bae et al. ** (2024) [19]	1078	Not reported	In HR− subtypes: calcifications are often benign; HR+ is more likely to have DCIS or minimal residual disease.	Residual calcifications in HR− rarely malignant; in HR+ may indicate DCIS; subtype-specific surgical tailoring advised.
Kim et al. (2024) [7]	323	38.5%	Outcomes driven by pCR rather than microcalcification persistence; molecular subtype influenced imaging accuracy.	Persistence of calcifications unrelated to recurrence; long-term outcome linked to pCR status, not imaging.

Notes: * Benign microcalcifications refer to those without residual invasive or in situ disease on pathology. ** This work is a conference abstract (SABCS), not peer-reviewed. ^†^ ICC—intraclass correlation coefficient.

**Table 2:** Comparative summary of studies about the accuracy of MRI and CEM in evaluating post-NAC microcalcifications.

Study (Year)	Number of Patients	Comparison: MG vs. MRI vs. CEM	Main Findings
Marinovich et al. (2013) [22]	2050	MRI had higher accuracy than mammography (*p* = 0.02); higher accuracy in studies that excluded residual DCIS from the definition of pCR (RDOR ^¥^ = 1.31, 95% CI = 0.33 to 5.20)	MRI remains the standard but is limited for DCIS; pooled MRI specificity declines when DCIS is included in the pCR definition.
Jochelson et al. (2015) [16]	111	Absence of MRI enhancement often indicated pCR; however, some non-enhancing DCIS persisted.	Non-enhancing areas on MRI are often benign; some DCIS are non-enhancing; MRI is not definitive.
An et al. (2017) [8]	29	MRI (CCC ^◊^ = 0.566) is better than MG (CCC ^◊^ = 0.196) in tumor size estimation.	44.8% of calcifications are benign; MRI moderately correlates with residual tumor size; MG overestimates.
Zhu et al. (2023) [27]	127	MRI showed a strong correlation with pathology (ICC ^†^ = 0.771); MG showed minimal correlation (ICC ^†^ = 0.097).	MRI is highly reliable for residual tumor extent; microcalcifications are not correlated with disease burden.
Um et al. (2018) [9]	151	MRI is more accurate than MG, especially in TNBC and HER2+ (ICC^†^ 0.939–0.543).	MRI is superior to MG, especially in TNBC and HER2+ tumors, and emphasizes the molecular subtype’s impact.
Barra et al. (2018) [29]	33	CEM sensitivity 93%; higher specificity (87.5%) and correlation with pathology than MRI (CCC ^◊^ 0.7 vs. 0.4).	CEM shows superior correlation with pathology and MG; it is reliable for visualizing microcalcifications.
Kim et al. (2020) [28]	96	MRI (ICC ^†^ = 0.709) is superior to MG (ICC ^†^ = 0.365); subtype-dependent accuracy.	MRI is the best predictor of residual extent; benign calcifications account for 38.5%; accuracy is subtype-dependent.
Iotti et al. (2017) [25]	46	Among patients with residual microcalcifications on MG after NAC, 40.9% (9/22) had no enhancement on CEM, and 77.8% of them (7/9) were confirmed as non-tumoral on final pathology	High negative predictive value of absent enhancement for excluding residual disease.
Savaridas et al. (2023) [26]	14	CEM (ICC ^†^ = 0.69) better correlated with DCIS extent than MRI (ICC ^†^ = 0.66).	CEM correlates better with DCIS and invasive tumor size and outperforms MRI.
Ploumen et al. (2023) [23]	1438	Up to 43% of DCIS lesions are non-enhancing on MRI, reducing sensitivity.	MRI misses non-enhancing DCIS in ~43% (lower pooled specificity, 0.61 versus 0.69); residual risk of underestimation.
Bernardi et al. (2022) [24]	51	CEM and MRI are highly concordant (difference < 1 mm relative to pathology); CEM is an alternative when MRI is contraindicated.	CEM and MRI are comparable in tumor sizing; CEM is a viable MRI alternative.
Patel et al. (2018) [30]	65	CEM and MRI have similar accuracy (±1 cm difference); CEM has a slightly higher PPV.	CEM has a slightly higher PPV; both are reliable for estimating residual disease.
Bae et al. * (2024) [19]	554	Complete MRI response predicted pCR (96% HR−, 64% HR+); residual microcalcifications are often benign.	MRI is highly predictive of pCR; benign microcalcifications are common in HR−.

Notes: * This work is a conference abstract (SABCS), not peer-reviewed. ^¥^ RDOR—relative diagnostic odds ratio. ^◊^ CCC—concordance correlation coefficient. ^†^ ICC—intraclass correlation coefficient.

## Data Availability

No new data were created or analyzed in this study.
